# Assessment of Unintentional Duplicate Orders by Emergency Department Clinicians Before and After Implementation of a Visual Aid in the Electronic Health Record Ordering System

**DOI:** 10.1001/jamanetworkopen.2019.16499

**Published:** 2019-12-02

**Authors:** Steven Horng, Joshua W. Joseph, Shelley Calder, Jennifer P. Stevens, Ashley L. O’Donoghue, Charles Safran, Larry A. Nathanson, Evan L. Leventhal

**Affiliations:** 1 Department of Emergency Medicine, Beth Israel Deaconess Medical Center, Boston, Massachusetts; 2 Division of Clinical Informatics, Beth Israel Deaconess Medical Center, Boston, Massachusetts; 3 Center for Healthcare Delivery Science, Beth Israel Deaconess Medical Center, Boston, Massachusetts

## Abstract

**Importance:**

Electronic health records allow teams of clinicians to simultaneously care for patients, but an unintended consequence is the potential for duplicate orders of tests and medications.

**Objective:**

To determine whether a simple visual aid is associated with a reduction in duplicate ordering of tests and medications.

**Design, Setting, and Participants:**

This cohort study used an interrupted time series model to analyze 184 694 consecutive patients who visited the emergency department (ED) of an academic hospital with 55 000 ED visits annually. Patient visits occurred 1 year before and after each intervention, as follows: for laboratory orders, from August 13, 2012, to August 13, 2014; for medication orders, from February 3, 2013, to February 3, 2015; and for radiology orders, from December 12, 2013, to December 12, 2015. Data were analyzed from April to September 2019.

**Exposure:**

If an order had previously been placed during the ED visit, a red highlight appeared around the checkbox of that order in the computerized provider order entry system.

**Main Outcomes and Measures:**

Number of unintentional duplicate laboratory, medication, and radiology orders.

**Results:**

A total of 184 694 patients (mean [SD] age, 51.6 [20.8] years; age range, 0-113.0 years; 99 735 [54.0%] women) who visited the ED were analyzed over the 3 overlapping study periods. After deployment of a noninterruptive nudge in electronic health records, there was an associated 49% decrease in the rate of unintentional duplicate orders for laboratory tests (incidence rate ratio, 0.51; 95% CI, 0.45-0.59), from 4485 to 2731 orders, and an associated 40% decrease in unintentional duplicate orders of radiology tests (incidence rate ratio, 0.60; 95% CI, 0.44-0.82), from 956 to 782 orders. There was not a statistically significant change in unintentional duplicate orders of medications (incidence rate ratio, 1.17; 95% CI, 0.52-2.61), which increased from 225 to 287 orders. The nudge eliminated an estimated 17 936 clicks in our electronic health record.

**Conclusions and Relevance:**

In this interrupted time series cohort study, passive visual cues that provided just-in-time decision support were associated with reductions in unintentional duplicate orders for laboratory and radiology tests but not in unintentional duplicate medication orders.

## Key Points

**Question:**

Can a simple visual aid reduce duplicate ordering in an electronic health record?

**Findings:**

This cohort study of 184 694 patients in an emergency department suggested that the introduction of a visual aid was associated with a 49% reduction in unintentional duplicate orders for laboratory tests and a 40% reduction in unintentional duplicate orders for radiology tests. There was no statistically significant change in unintentional duplicate orders for medications.

**Meaning:**

The results of this study suggest that a passive visual aid that guides clinicians to the right action is a useful alternative to an interruptive alert.

## Teaser

This cohort study determines whether a simple visual aid is associated with a reduction in duplicate ordering of tests and medications in an emergency department.

## Introduction

Electronic health records (EHRs) could improve patient safety by facilitating communication, providing access to information, assisting with calculations, monitoring patients, providing decision support, and enhancing clinician situational awareness.^1,2^ However, the very presence of EHRs may lead to unintended consequences, such as increasing the likelihood that health care professionals will overlook existing orders and duplicate work.^3,4^ Duplicate orders may be the intention of a clinician looking to measure a laboratory value or radiology study serially. However, duplicate orders may also be markers of poor communication between clinicians caring for the same patient or may indicate that an order has been placed for the wrong patient.^5^ If unrecognized, these duplicate orders could lead to patient harm. If intercepted, these near-misses produce unnecessary work.

Common strategies to reduce duplicate orders include additional training for users, downstream workflow mitigation (such as screening by pharmacy, laboratory, or radiology departments), or interruptive alerts.^6^ Although interruptive alerts can be effective, they generally occur after the clinician has completed the ordering process, which can result in increased click count, cognitive dissonance, frustration, and alert fatigue.^7,8^ Interruptive alerts can also disrupt thought processes, which may lead to more errors.^9,10,11,12,13^ Furthermore, poor EHR design and use factors have been associated with clinician stress and burnout.^14^

Weed, an early pioneer of EHRs and inventor of the SOAP (ie, subjective, objective, assessment, plan) note, proposed a different approach.^15^ He argued that EHRs could both guide and teach.^16^ Passive just-in-time (JIT) reminders help guide users to the right action rather than preventing them from taking the wrong action. Passive reminders are less disruptive than interruptive alerts. The level of disruption to clinician workflows should be tailored to the severity and immediacy of the harm being prevented.^17^

The time-sensitive nature of emergency department (ED) visits, combined with the need for parallel workflows and team-based care, makes the ED setting particularly vulnerable to unintended duplicate orders.^18,19^ The goal of this investigation was to assess unintentional duplicate orders in the ED after implementation of passive, inline, JIT decision support, which functioned as a nudge for clinicians.

## Methods

### Study Design

We performed an interrupted time series analysis cohort study to analyze all patient visits 1 year before and 1 year after each intervention. As this was a retrospective analysis of a quality improvement project, a determination was made by the Beth Israel Deaconess Medical Center Committee on Clinical Investigation that this did not constitute human subjects research and no further review or approval was required. This study followed the Strengthening the Reporting of Observational Studies in Epidemiology (STROBE) reporting guideline.

### Setting and Selection of Participants

The study was performed in a level I trauma center and tertiary, academic, adults-only, teaching ED with 55 000 annual visits. We performed a 3-phase implementation of the intervention. All consecutive ED patient visits 1 year before and 1 year after each intervention were included in the study. As the intervention dates were different, the periods of measurement were different for each intervention, as follows: for laboratory orders, from August 13, 2012, to August 13, 2014; for medication orders, from February 3, 2013, to February 3, 2015; and for radiology orders, from December 12, 2013, to December 12, 2015. No visits were excluded. The EHR used was developed at the institution.

### Intervention

**Figure 1.**
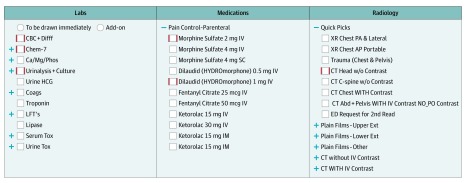
Passive, Inline, Just-in-Time Duplicate Order Decision Support A red highlight was placed around the checkbox if the order had previously been placed during the same emergency department visit.

We implemented a user interface within our computerized provider order entry (CPOE) system in the ED that provided passive, inline, JIT duplicate order decision support while the user was in the process of placing an order. If an order had previously been placed during that ED visit, the user was cued by a red highlight around the checkbox of that order (Figure 1). The intervention was performed as a phased implementation, starting with laboratory orders (August 13, 2013), followed by medication orders (February 3, 2014) and radiology orders (December 12, 2014).

### Duplicate Order Algorithm

If 2 identical orders were placed and 1 was subsequently cancelled during that ED visit, the second order was identified as a duplicate. For the purposes of this algorithm, a laboratory order was uniquely identified by an internal laboratory code, which is mapped to a Logical Observation Identifiers Names and Codes code. Medication orders were uniquely identified by the combination of an internal medication code, route, and dose. For example, 2 orders for 5 mg of intravenous morphine once would be considered a duplicate order, while an order for 5 mg of intravenous morphine once and 2 mg of intravenous morphine once would not be considered a duplicate. Each internal medication code is mapped to a medication entry in First Databank. Radiology orders were uniquely identified by a Simon-Leeming code.^20^

### Methods of Measurement

The unit of analysis was at the shift level to adjust for environmental variables, such as team workload, team communication, and other team dynamics that might place the patient in a risky state^21^ and increase the risk of a duplicate order. Clinicians work 8-hour shifts (ie, 7 am-3 pm, 3 pm-11 pm, and 11 pm-7 am). We identified the patients who arrived in the ED during each shift. For each patient, we collected the following environmental variables at the patient’s time of arrival: current number of patients in the ED, number of patients in observation, number of new patients, number of intensive care unit (ICU) bed requests, number of telemetry bed requests, number of floor bed requests, and number of boarders (ie, patients who have been admitted to the hospital but have not received a bed after 2 hours). We then calculated the mean for each environmental variable for each shift. For each shift, we also calculated mean patient age, percentage of women patients, percentage of patients with an emergency severity index of at least 3 (range, 1-5; with 1 indicating a critically ill patient requiring immediate intervention and 5 indicating a patient with the least acuity), percentage of patients with English as a primary language, percentage of patients discharged, percentage of patients admitted, and length of stay in the ED. Because orders are generally placed during the first part of a patient’s visit, patients are assigned to a shift based on their time of arrival rather than a weighted representation during their entire visit. The outcome measure was the number of unintentional duplicate orders for each shift for each order type, ie, laboratory, medication, and radiology.

### Statistical Analysis

Means with SDs were reported for continuous variables, while counts and percentages were reported for categorical variables. Univariate significance testing was performed using *t* tests for continuous variables and χ^2^ tests for categorical variables.

For the main outcome, data were analyzed using an interrupted time series analysis at the shift level. We performed individual negative binomial models for each of the duplicate order types, ie, laboratory orders, medication orders, and radiology studies. The natural log of the total number of orders was included as an offset term. All models were adjusted at the shift level by mean patient age, sex composition of patients, percentage of patients who were native English speakers, percentage of patients with an emergency severity index of 3 or greater, percentage of patients discharged home, percentage of patients admitted, number of patients in the ED, number of patients in the waiting room, number of observation patients, number of new patients, number of ICU beds requested, number of telemetry beds requested, number of boarders, and length of stay in the ED. Full sets of indicator variables were included for time of day, day of week, and month at the shift level because duplicate orders may follow a cyclical pattern throughout the day, week, and year, respectively. We reported both the immediate effect size, or the level change, of the intervention on duplicate orders and an estimate of the change in trend of duplicate orders after the intervention.

Coefficients were transformed into incidence rate ratios (IRRs) with 95% CIs. *P* ≤ .05 was considered statistically significant, and all tests were 2-tailed. Stata SE version 14.2 was used for statistical analysis (StataCorp). This analysis was performed from April 2019 to September 2019. A more detailed description of the statistical analysis can be found in the eMethods in the Supplement.

## Results

### Summary Statistics

Data on 184 694 ED patients were collected during 1217 days from August 13, 2012, to December 12, 2015, through 3 overlapping study periods. Patient mean (SD) age was 51.6 (20.8) years (range, 0-113.0 years), and there were 99 735 women (54.0%) across all study periods. The laboratory study period analyzed 110 227 patients from August 13, 2012, to August 13, 2014, with 4485 unintentional duplicate laboratory orders in the year before the intervention and 2731 in the year after the intervention. The medication study period analyzed 110 202 patients from February 3, 2013, to February 3, 2015, with 225 unintentional duplicate orders in the year before the intervention and 287 in the year after the intervention. The radiology study period analyzed 111 414 patients from December 12, 2013, to December 12, 2015, with 956 unintentional duplicate orders in the year before the intervention and 782 in the year after the intervention. Patient demographic characteristics and order summary statistics are reported in Table 1.

**Table 1.** Patient Demographic Characteristics and Order Summary Statistics^a^

Characteristic	Mean (SD)								
	Laboratory			Medications			Radiology		
	Before Intervention (n =55 018)	After Intervention (n = 55 227)	*P* Value	Before Intervention (n = 54 475)	After Intervention (n = 55 750)	*P* Value	Before Intervention (n = 55 302)	After Intervention (n = 56 128)	*P* Value
Patient age, y	51.16 (20.74)	51.75 (20.75)	<.001	51.21 (20.75)	51.85 (20.74)	<.001	51.40 (20.75)	51.92 (20.75)	<.001
Female patients, No. (%)	29 936 (54.4)	30 067 (54.4)	.92	29 530 (54.2)	30 496 (54.7)	.10	30 284 (54.8)	30 503 (54.3)	.16
Patients with ESI ≥3, No. (%)	33 996 (61.8)	33 597 (60.8)	.001	33 559 (61.6)	33 483 (60.1)	<.001	33 350 (60.3)	32 685 (58.2)	<.001
Patients with English as primary language, No. (%)	53 069 (96.5)	53 205 (96.3)	.29	52 499 (96.4)	53 612 (96.2)	.07	53 196 (96.2)	54 052 (96.3)	.34
Discharged, No. (%)	32 317 (58.7)	32 391 (58.7)	.77	32 007 (58.8)	32 186 (57.7)	.001^a^	32 023 (57.9)	32 227 (57.4)	.10
Admitted, No. (%)	20 259 (36.8)	20 312 (36.8)	.44	20 149 (37.0)	20 848 (37.4)	.16	20 613 (37.3)	20 913 (37.3)	.96
Patients in department, No.	41.21 (10.90)	44.79 (11.11)	<.001	40.93 (10.85)	45.89 (10.93)	<.001	42.14 (10.99)	47.35 (10.72)	<.001
Patients in waiting room, No.	4.47 (4.90)	5.71 (5.81)	<.001	4.46 (4.95)	6.02 (5.94)	<.001	4.74 (5.14)	6.71 (6.27)	<.001
Patients in observation, No.	8.69 (4.16)	8.95 (4.35)	<.001	8.44 (4.12)	9.21 (4.40)	<.001	8.53 (4.17)	9.65 (4.46)	<.001
New patients, No.	7.96 (3.70)	7.93 (3.66)	.14	7.84 (3.69)	7.99 (3.67)	<.001	7.91 (3.68)	8.00 (3.67)	<.001
ICU bed requests, No.	0.85 (1.00)	1.10 (1.15)	<.001	0.87 (1.00)	1.15 (1.18)	<.001	0.91 (1.03)	1.30 (1.24)	<.001
Telemetry bed requests, No.	2.51 (2.09)	3.44 (2.59)	<.001	2.52 (2.08)	3.66 (2.66)	<.001	2.77 (2.26)	4.06 (2.74)	<.001
Floor bed requests, No.	3.67 (2.59)	4.30 (2.88)	<.001	3.56 (2.55)	4.53 (2.93)	<.001	3.76 (2.64)	4.92 (3.03)	<.001
Boarders, No.	2.72 (2.48)	2.33 (2.69)	<.001	2.18 (2.36)	2.65 (2.81)	<.001	2.06 (2.33)	3.33 (3.05)	<.001

Abbreviations: ESI, emergency severity index; ICU, intensive care unit.

^a^ Patient demographics and order summary are reported at the patient level.

### Main Results

**Figure 2.**
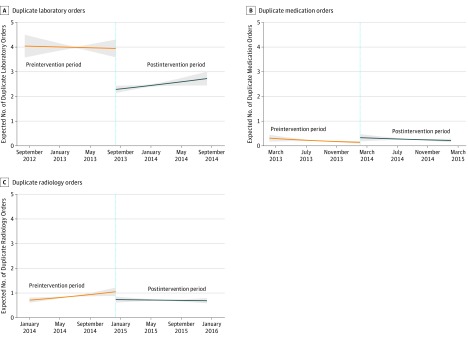
Interrupted Time Series of Unintentional Duplicate Orders Shaded areas indicate 95% CIs.

After the intervention, there was a significant level change with an associated decrease in laboratory duplicate orders (IRR, 0.51; 95% CI, 0.45-0.59) and associated decrease in radiology duplicate orders (IRR 0.60; 95% CI, 0.44-0.82). Additionally, while the estimate for medication duplicate orders was positive, it was not statistically significant, with an IRR of 1.17 (95% CI, 0.52-2.61). There was no statistically significant change in trend at the 5% significance level for any of the intervention groups. We report these results from the interrupted time series in Table 2 and Figure 2. The full results of all covariates in the model can be found in eTable 1 in the Supplement.

**Table 2.** Unadjusted Results and Interrupted Time Series Analysis

Setting	Mean (SD)			IRR (95% CI)		
	Orders, No.	Duplicate Orders, No.	Proportion Duplicate Orders	Level Change^a^	Change in Trend^b^	Preintervention Trend^c^
**Laboratory**						
Before intervention	6.10 (6.13)	0.08 (0.63)	0.01 (0.05)	0.51^a^ (0.45-0.59)	1.00 (1.00-1.00)	1.00 (1.00-1.00)
After intervention	6.21 (5.95)	0.05 (0.48)	0.01 (0.04)			
*P *value	.001	<.001	<.001	<.001	<.001	<.001
**Medication**						
Before intervention	2.81 (3.69)	0.00 (0.07)	0.00 (0.01)	1.17 (0.52-2.61)	1.00 (1.00-1.00)	1.00 (1.00-1.00)
After intervention	3.12 (4.03)	0.01 (0.08)	0.00 (0.01)			
*P *value	<.001	.002	.02	.71	<.001	.08
**Radiology**						
Before intervention	1.12 (1.43)	0.02 (0.15)	0.01 (0.05)	0.61 (0.46-0.82)	1.00 (1.00-1.00)	1.00 (1.00-1.00)
After intervention	1.18 (1.48)	0.01 (0.13)	0.01 (0.05)			
*P *value	<.001	.02	.002	.001	<.001	.007

Abbreviation: IRR, incidence rate ratio.

^a^ Estimates the immediate change in the incidence of duplicate orders after implementation.

^b^ Estimates the gradual change in the incidence of duplicate orders over time after implementation.

^c^ Estimates the preintervention trend in incidence over time.

In our EHR, it takes a minimum of 9 clicks and password entry to cancel an order. Estimating the burden of order cancellation at 9 clicks and 30 seconds, the estimated reduction in unintended duplicate orders saved 17 936 clicks (not including the password) in the year after the intervention, which amounts to 16 hours and 36 minutes of regained productivity.

For patient variables, an emergency severity score of at least 3 (ie, lower acuity) was associated with more radiology duplicate orders after the intervention (IRR, 1.01; 95% CI, 1.00-1.01) but fewer medication duplicate orders (IRR, 0.99; 95% CI, 0.99-0.99). For environmental variables, the number of patients in the waiting room was associated with an increase in medication duplicate orders (IRR, 1.05; 95% CI, 1.04-1.07) and radiology duplicate orders (IRR, 1.01; 95% CI, 1.00-1.02). The number of new patients during a shift was associated with a decrease in laboratory duplicate orders (IRR, 0.97; 95% CI, 0.96-0.99) and medication duplicate orders (IRR, 0.83; 95% CI, 0.79-0.88). The number of patients admitted to the ICU during a shift was associated with a decrease in the number of laboratory duplicate orders (IRR, 0.95; 95% CI, 0.96-0.99). The number of patients admitted to the floor during a shift was associated with a decrease in laboratory duplicate orders (IRR, 0.97; 95% CI, 0.96-0.99) and an increase in radiology duplicate orders (IRR, 1.03; 95% CI, 1.01-1.05). The number of patients boarding during a shift was associated with an increase in laboratory duplicate orders (IRR, 1.05; 95% CI, 1.03-1.08).

The most commonly duplicated laboratory order was the basic metabolic panel; the most commonly duplicated medication was intravenous hydromorphone; and the most commonly duplicated radiology test was chest radiography. The 5 most commonly duplicated orders for each order type are reported in eTable 2 in the Supplement.

## Discussion

### Just-in-Time Passive Decision Support

In our ED, adding a red highlight around an order’s checkbox (Figure 1) was associated with a significant reduction in duplicate orders for both laboratory and radiology testing but not medication. We demonstrated a simple yet useful method that was associated with a reduction in duplicate laboratory and radiology orders that avoids many of the downsides of traditional, interruptive methods. Our method was evaluated in a busy clinical setting that relies on effective and efficient teamwork to care for patients. The intervention resulted in a time savings of 16 hours and 36 minutes for clinicians. This estimate may lack generalizability because different EHRs will have different order cancellation workflows that may be more or less burdensome.

We followed the best practices of clinical decision support to minimize interruptions unless they were clinically necessary.^17^ We placed the decision support for duplicate orders JIT, which is exactly when the decision to order was being made, rather than using an interruptive alert, which appears after a decision has already been made. We also placed our decision support inline, so that the decision support was in the same area of focus as the intended action. This is in contrast to the ASAP ER Module (Epic Systems), in which a passive reminder appears in the order queue. Although similar, the duplicate order decision support in the ASAP ER module is not delivered until after the user has selected the order. Ideally, decision support should appear before the user has selected the order.

Unlike laboratory and radiology duplicate orders, we were unable to detect a difference in medication duplicate orders. At baseline, we had relatively few medication duplicate orders compared with laboratory and radiology duplicate orders, which is consistent with ED workflow. Nurses often order laboratory and radiographic studies per clinical protocol but do not routinely order medications, except in an emergency when a physician gives a nurse a verbal order. A medication duplicate order would normally only occur if 2 physicians (eg, a resident and an attending) both ordered the same medication.

### Comparison With Interruptive Alerts

Interruptive alerts fundamentally make the wrong action hard to do but often with substantial cost to physician time. In a CPOE implementation in the ICU setting^4^ using EpicCare Inpatient Clinical System (Epic Systems), duplicate order decision support was implemented as a postorder interruptive alert. It was thought that the poor alert design interface and high false-positive rate led to alert fatigue and frequent overrides, with some overrides occurring without the user reading the alert.^4^

In contrast to our strategy, Procop et al^22^ used a hard-stop phone call, which required physicians to call the laboratory to order a duplicate from a list of more than 1200 lab tests. This intervention was associated with a reduction in duplicate lab orders, with only 3% of interruptive alerts being overridden by a phone call, and resulted in a cost avoidance of $183 586 during 2 years, which accounted for materials and laboratory personnel labor but failed to account for physician labor used to initiate the call. Although poor usability can be a powerful deterrent, we believe good usability is a more sustainable approach that respects human autonomy and promotes user satisfaction and physician longevity.

Not only do interruptive alerts increase cognitive burdens and disrupt workflows, but they can also lead to delays in treatment. In 2006, a multicenter randomized clinical trial^23^ was performed to evaluate an interruptive alert to reduce concomitant orders for warfarin and trimethoprim-sulfamethoxazole. Although the interruptive alert was successful in reducing ordering of this drug pair, with an adjusted odds ratio of 0.12 (95% CI, 0.05-0.33), it had the unintended consequence of delays in treatment for 4 patients. The harm was believed sufficient for the institutional review board to terminate the study early.

### Additional Use Cases

**Figure 3.**
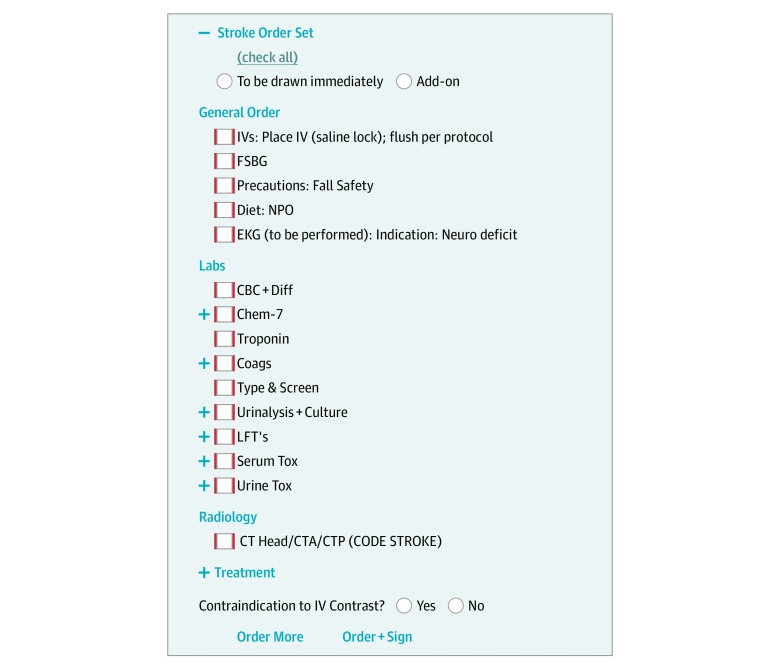
Order Sets as Visual Checklists

We used the same techniques for duplicate order reminders in our order sets, transforming the order set itself into a visual checklist, as shown in Figure 3. These visual checklists engaged the visual and spatial reasoning portions of the brain, offloading the language centers. These same techniques could also be used to guide users when using clinical decision rules, as shown in eFigure 1 in the Supplement, suggesting the correct responses based on automated information retrieval. In the same way, such methods could also be used to integrate predictive machine learning algorithms into clinical workflows by guiding users to the correct response along with some explanation from the algorithm to show its work. Since a human is still in the loop, this method would be tolerant to less-than-perfect performance metrics as newer machine learning models are being created.

Although for this application we used passive, inline, JIT decision support alone, this approach need not be mutually exclusive with interruptive alerts. For example, we have also implemented common drug decision support in CPOE and prescribing modules for allergies, pregnancy, and drug-drug interactions using both approaches. Allergy decision support for CPOE and prescribing modules is very amenable to this approach because some patients can have multiple drug allergies, shown in eFigure 2 in the Supplement. Although EHRs commonly provide JIT decision support by enumerating a patient’s allergies on the order screen, users must commit these allergies to working memory and then apply them by drug class to each potential order. This common workflow creates multiple points of failure: committing allergies to working memory, grouping allergies to drug classes, and cross-referencing drug classes to orders. Our inline decision support offloads this cognitive process from the user, freeing their attention to focus on other clinical tasks.

### Future Directions

Our implementation was specific to the ED, but is applicable to any care setting where clinical care teams must collaborate. Other care settings that have clinicians who are geographically dispersed would amplify the changes associated with such an intervention. Different care settings would require different thresholds for determining if an order is a duplicate. For example, in the ICU, that threshold might be 4 to 12 hours. In the hospitalist setting, that threshold may be 24 hours, while in the outpatient setting, it may be 1 week or 1 month. In the ED, having a simple binary representation of whether an order was a duplicate during that ED visit was intuitive and clinically meaningful. However, in the ICU, having multiple stages corresponding with last order time with additional information, such as ordered 5 minutes ago or ordered 6 hours ago, could be important. We plan to iteratively develop new user interfaces given this increased complexity as we deploy this intervention across different care settings.

### Limitations

This study had limitations. First, we conducted an observational study, and our results may be the consequence of unmeasured confounding variables. Although randomized clinical trials remain the criterion standard for assessing the effect of interventions, it is not always possible in informatics-based interventions in the ED. To mitigate the risks of observational design, we performed an interrupted time series analysis and adjusted for every patient-level variable or environmental variable collected in our EHR that might be associated with duplicate orders. Furthermore, in our intervention, the change in the number of orders was unlikely owing to chance or secular trends because the individual interventions on laboratory, medication, and radiology orders were phased in at different times. In our interrupted time series analysis, the unit of analysis was at the level of the shift. Alternatively, we could have elected not to perform a time series analysis at all and instead performed a before-and-after study at the order or patient level. However, the advantage of an interrupted time series analysis is that it can help distinguish the change of an intervention from a preexisting trend; however, it requires aggregating patients into time points. We chose a unit of analysis at the shift level to adjust for environmental variables that might place the patient in a risky state^21^ and increase the risk of a duplicate order. For example, a crowded ED with a large number of patients and boarders likely puts stress on the care team and increases the risk of errors. Rather than adjust for variables that put an individual patient at risk, we adjusted for variables that put the system at risk, a different paradigm in patient safety.

Second, our outcome measure only captured duplicate orders that were subsequently cancelled, using the act of cancellation as a surrogate for user intent. There were also unintentional duplicate orders that were never cancelled and subsequently carried out.

Third, our intervention and outcome measure only addressed exact duplicate orders. Semantic duplicate orders, such as therapeutic duplication, are also important and remain as future work. For example, a clinician who ordered acetaminophen/oxycodone should receive passive JIT decision support not only for acetaminophen/oxycodone but also for its constituent ingredients, ie, acetaminophen and oxycodone.

Fourth, our study was performed at an urban, academic, adults-only teaching hospital with a custom EHR. The usefulness of this intervention may differ in other care settings with different baseline unintentional duplicate orders and mitigation workflows. Furthermore, our clinical leadership team decided that 2 identical orders placed during the same ED visit could be a potential duplicate order. However, other EDs with substantial boarding times or different workflows would require different decision support triggering thresholds.

## Conclusions

In this cohort study, passive visual cues that provided JIT decision support to clinicians were associated with reductions in unintentional duplicate orders for laboratory and radiology tests but not for medication. This type of EHR-based reminder may be a useful alternative to interruptive, postorder alerts for reducing duplicate order entry. We believe guiding clinicians to a right action is better than telling the clinician they have made an error. This approach may help reduce alert fatigue and lessen clinician stress and burnout associated with EHRs.

## Notes

Supplement. **eMethods.** Statistical Methods **eTable 1. **Interrupted Time Series Analysis, Full Covariate List **eTable 2. **Top 5 Unintentional Duplicate Orders **eFigure 1. **Visual Aid as Suggestion for Clinical Decision Rules **eFigure 2. **Visual Aid for Allergy Decision Support
