# Supplementary material for: Assessment of Unintentional Duplicate Orders by Emergency Department Clinicians Before and After Implementation of a Visual Aid in the Electronic Health Record Ordering System
Source: JAMA Netw Open. 2019 Dec 2;2(12):e1916499. doi: 10.1001/jamanetworkopen.2019.16499 (PMC6902748; doi:10.1001/jamanetworkopen.2019.16499)
Supplement: Supplement. — eMethods. Statistical Methods eTable 1. Interrupted Time Series Analysis, Full Covariate List eTable 2. Top 5 Unintentional Duplicate Orders eFigure 1. Visual Aid as Suggestion for Clinical Decision Rules eFigure 2. Visual Aid for Allergy Decision Support [file jamanetwopen-2-e1916499-s001.pdf]

Supplementary Online Content

Horng S, Joseph JW, Calder S, et al. Assessment of unintentional duplicate orders by emergency department clinicians before and after implementation of a visual aid in the electronic health record ordering system. *JAMA Netw Open*. 2019;2(12):e1916499. doi:10.1001/jamanetworkopen.2019.16499

eMethods. Statistical Methods
eTable 1. Interrupted Time Series Analysis, Full Covariate List
eTable 2. Top 5 Unintentional Duplicate Orders
eFigure 1. Visual Aid as Suggestion for Clinical Decision Rules
eFigure 2. Visual Aid for Allergy Decision Support

This supplementary material has been provided by the authors to give readers additional information about their work.

© 2019 Horng S et al. *JAMA Network Open.*

eMethods. Statistical Methods

We modeled duplicate orders for each of the three order types with the following negative binomial regression:

$$ln(\mu) = \beta_0 + \beta_1 Post_t + \beta_2(Post_t * T_t) + \beta_3 T_t + \beta_4 X_t$$

where μ represents the expected number of duplicate orders of its type. The natural log of the total number of orders were included as an offset term. $Post_t$ is an indicator variable that equals 1 after the intervention date for that order type and 0 before. T_t is the number of shifts at time *t* since the beginning of the sample for that order type, where the sample begins 1 year prior to the intervention date. X_t is a vector of covariates at time *t* at the shift-level, including average patient age, gender composition of patients, whether patients were native English speakers, percent of patients with an emergency severity index 3 or greater, percent of patients discharged, percent of patients admitted, number of patients in the department, number of patients in the waiting room, number of observation patients, number of new patients, number of ICU beds requested, number of telemetry beds requested, number of boarders, length of stay in the emergency department. Full sets of indicator variables for shift, day-of-week, and month-of-sample are included to adjust for how duplicate orders may follow a cyclical pattern throughout the day, week, and year, respectively. We estimate Newey-West standard errors, which are robust to autocorrelation and heteroskedasticity. All tests used a significance level of 0.05.

Coefficients are transformed into incidence rate ratios (IRRs) in Table 2, such that e^{β_1} estimates the immediate change in the incidence of duplicate orders after implementation, e^{β_2} estimates the gradual change in the incidence of duplicate orders over time after implementation, e^{β_3} estimates the pre-intervention trend in incidence over time.

© 2019 Horng S et al. *JAMA Network Open.*

We initially used a Poisson regression to model counts. However, a likelihood ratio test of alpha = 0 generated a large test statistic of 2734.83 with an associated p-value of < 0.001. This suggests that our duplicate order response variables are over-dispersed and therefore we used the more general negative binomial model.

Because we estimated three separate models for each of the types of duplicate orders, the coefficients on the covariates are estimated separately. One may be concerned that the sign and magnitude of these covariates should not be different across the three models. For instance, the association between average age and the probability of duplicate orders should likely be the same across the three types of orders as age should not be associated with the probability of a laboratory duplicate order differently than a medication or radiology duplicate order. Indeed, when testing the equality of the coefficients on the covariates across the three models, we find that they are not statistically different from each other, with the only exception being number of patients in the waitroom. Thus, we re-estimated our models with the imposed constraint that the estimated coefficients on the covariates that were not statistically different from each other are equal across models. This allows us to estimate the association between the intervention and unintentional duplicate orders of each of the order types while jointly estimating the coefficients on the covariates overall. In this alternative specification, our estimates do not change in magnitude, direction, or significance.

Stata/SE 14.2 software was used for statistical analysis.

© 2019 Horng S et al. *JAMA Network Open.*

eTable 1. Interrupted Time Series Analysis, Full Covariate List

Variable	Laboratory	Medications	Radiology
	IRR[a] (95% CI[b])	IRR[a] (95% CI[b])	IRR[a] (95% CI[b])
Level Change[d]	0.51[c] (0.45-0.59)	1.17 (0.52-2.61)	0.61[c] (0.46-0.82)
Change in Trend[e]	1.00[c] (1.00-1.00)	1.00[c] (1.00-1.00)	1.00[c] (1.00-1.00)
Pre-Intervention Trend[f]	1.00 (1.00-1.00)	1.00 (1.00-1.00)	1.00 (1.00-1.00)
Age	1.00 (0.99-1.01)	0.99 (0.98-1.00)	1.00 (0.99-1.01)
Gender (Female)	1.00 (1.00-1.01)	1.00 (0.99-1.02)	1.00 (0.99-1.00)
Emergency Severity Index >= 3	1.00 (1.00-1.00)	0.99[c] (0.99-0.99)	1.01[c] (1.00-1.01)
Primary Language (English)	1.00 (0.99-1.01)	1.00 (0.98-1.03)	0.99 (0.98-1.00)
Patients Discharged Home	1.01 (0.99-1.03)	1.00 (0.99-1.01)	1.01 (1.00-1.02)
Patients Admitted	1.01 (0.99-1.02)	1.00 (0.99-1.02)	1.01[c] (1.00-1.01)
No. Patients in Department	1.00 (0.98-1.03)	0.99 (0.97-1.01)	1.00 (0.99-1.00)
No. Patients in Waitroom	0.99 (0.98-1.00)	1.05[c] (1.04-1.07)	1.01[c] (1.00-1.02)
No. Patients in Observation	1.01 (0.99-1.02)	1.00 (0.95-1.05)	1.02 (1.00-1.04)
No. New Patients	0.97[c] (0.96-0.99)	0.83[c] (0.79-0.88)	0.99 (0.94-1.03)
No. Patients Admitted to ICU	0.95[c] (0.90-0.99)	1.02 (0.93-1.11)	1.04 (1.00-1.08)
No. Patients Admitted to Telemetry	0.99 (0.95-1.04)	1.02 (1.00-1.04)	1.01 (0.98-1.03)
No. Patients Admitted to Floor	0.97[c] (0.96-0.99)	1.01 (0.96-1.07)	1.03[c] (1.01-1.05)
No. Patients Boarding	1.05[c] (1.03-1.08)	0.99 (0.97-1.02)	1.01 (0.99-1.02)
Length of Stay	1.00 (1.00-1.00)	1.00 (1.00-1.00)	1.00 (1.00-1.00)

Interrupted time series analysis was performed using a negative binomial regression with Newey-West standard errors at the shift level. All models contain a full set of indicator variables for time of day at the shift level, day-of-week, and month-of-sample.

[a] IRR = Incidence Rate Ratios

[b] 95% CI = 95% Confidence Intervals

© 2019 Horng S et al. *JAMA Network Open.*

[c] Denotes p-value < 0.05
[d] Estimates the immediate change in the incidence of duplicate orders after implementation.
[e] Estimates the gradual change in the incidence of duplicate orders over time after implementation.
[f] Estimates the pre-intervention trend in incidence over time.

© 2019 Horng S et al. *JAMA Network Open.*

eTable 2. Top 5 Unintentional Duplicate Orders

	Laboratory Duplicate Orders			
	BEFORE		AFTER	
**Laboratory Test**	Rank	Count (%)*	Rank	Count (%)[a]
Chem-7	1	726 (16%)	1	361 (13%)
CBC/DIF	2	615 (13%)	2	269 (10%)
Coags	3	239 (5%)	4	171 (6%)
Urinalysis	4	197 (4%)	11	76 (3%)
Lactate	5	194 (4%)	6	124 (5%)
	. . .		. . .	
	**Total**	**4560**	**Total**	**2729**
	**Medication Duplicate Orders**			
	BEFORE		AFTER	
**Medication Name**	Rank	Count (%)*	Rank	Count (%)[a]
Hydromorphone (IV)	1	53 (14%)	1	58 (12%)
Diazepam (PO)	2	33 (9%)	2	42 (8%)
Morphine (IV)	3	26 (7%)	3	29 (6%)
Lorazepam (IV)	4	11 (3%)	9	11 (2%)
Aspirin (PO)	5	8 (2%)	33	3 (1%)
	. . .		. . .	
	**Total**	**380**	**Total**	**503**
	**Radiology Duplicate Orders**			
	BEFORE		AFTER	

© 2019 Horng S et al. *JAMA Network Open.*

**Radiology Test**	Rank	Count (%)*	Rank	Count (%)[a]
Chest X-Ray (PA & Lat)	1	149 (15%)	2	101 (13%)
CT Head w/o Contrast	2	118 (12%)	3	86 (11%)
CT Abdomen & Pelvis w/ Contrast	3	113 (12%)	1	117 (15%)
CT C-Spine w/o Contrast	4	45 (5%)	7	26 (3%)
Chest X-Ray (Portable)	5	43 (4%)	4	34 (4%)
	. . .		. . .	
	**Total**	**962**	**Total**	**791**

[a] Count (%) should only be compared vertically, not horizontally, as count (%) are not normalized for differences in the number of patients and total orders in each period. Please use the ranks to compare horizontally. Please refer to *Table 1* and *Table 2* for normalized results to compare between periods.

© 2019 Horng S et al. *JAMA Network Open.*

eFigure 1. Visual Aid as Suggestion for Clinical Decision Rules

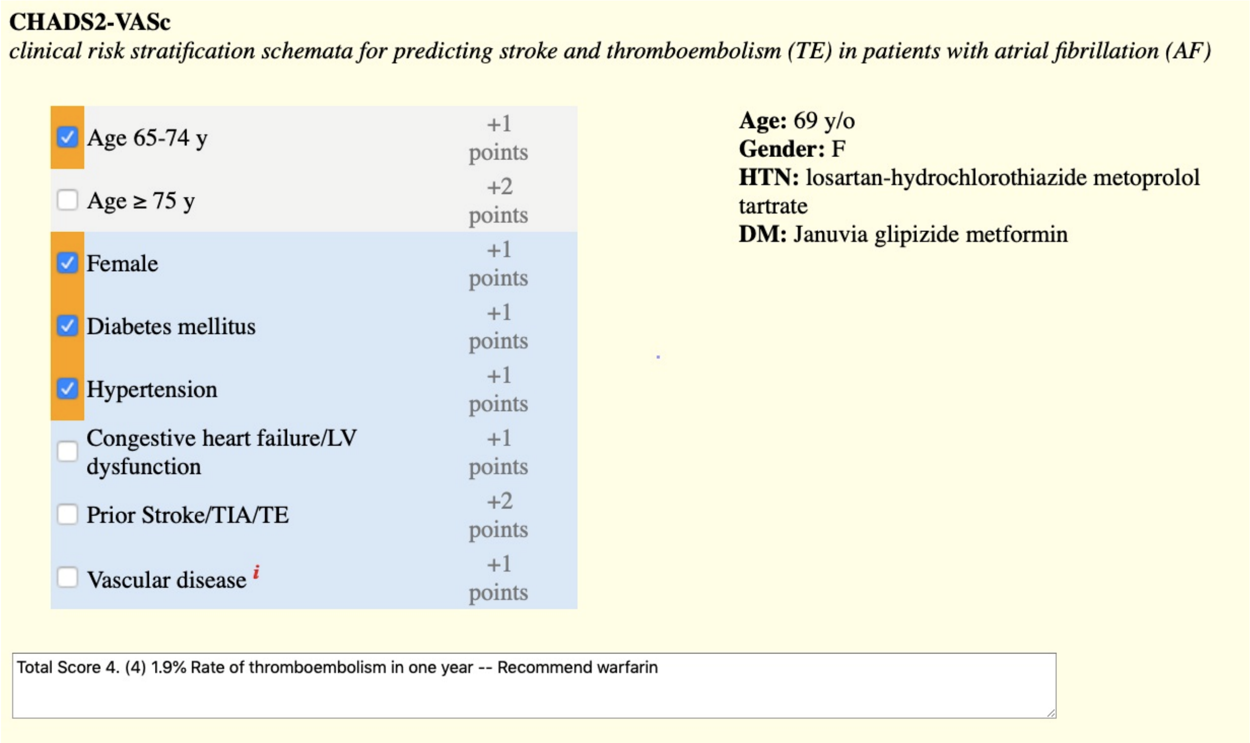

© 2019 Horng S et al. *JAMA Network Open.*

eFigure 2. Visual Aid for Allergy Decision Support

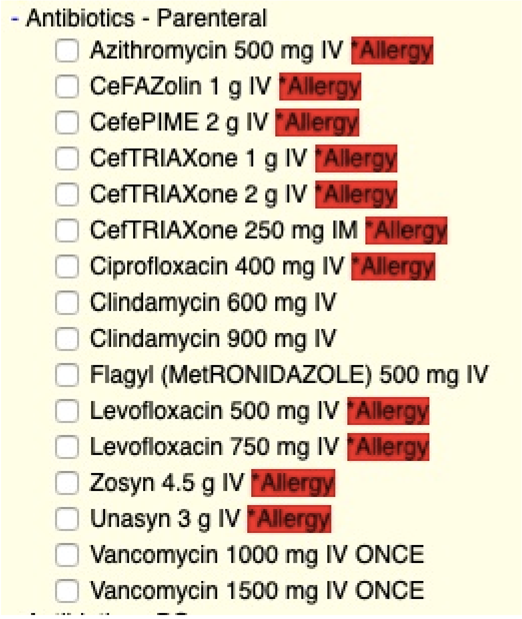

© 2019 Horng S et al. *JAMA Network Open.*
